# Evaluating the lethal and pre-lethal effects of a range of fungi against adult *Anopheles stephensi* mosquitoes

**DOI:** 10.1186/1475-2875-11-365

**Published:** 2012-11-05

**Authors:** Simon Blanford, Nina E Jenkins, Andrew F Read, Matthew B Thomas

**Affiliations:** 1 Center for Infectious Disease Dynamics, Department of Biology, Penn State University Mueller Laboratory, University Park, PA, 16802, USA; 2 Center for Infectious Disease Dynamics, Department of Entomology, Penn State University Merkle Lab, University Park, PA, 16802, USA; 3 Fogarty International Center, National Institutes of Health, Bethesda, MD, 20892, USA

## Abstract

**Background:**

Insecticide resistance is seriously undermining efforts to eliminate malaria. In response, research on alternatives to the use of chemical insecticides against adult mosquito vectors has been increasing. Fungal entomopathogens formulated as biopesticides have received much attention and have shown considerable potential. This research has necessarily focused on relatively few fungal isolates in order to ‘prove concept’. Further, most attention has been paid to examining fungal virulence (lethality) and not the other properties of fungal infection that might also contribute to reducing transmission potential. Here, a range of fungal isolates were screened to examine variation in virulence and how this relates to additional pre-lethal reductions in feeding propensity.

**Methods:**

The Asian malaria vector, *Anopheles stephensi* was exposed to 17 different isolates of entomopathogenic fungi belonging to species of *Beauveria bassiana*, *Metarhizium anisopliae*, *Metarhizium acridum* and *Isaria farinosus*. Each isolate was applied to a test substrate at a standard dose rate of 1×10^9^ spores ml^-1^ and the mosquitoes exposed for six hours. Subsequently the insects were removed to mesh cages where survival was monitored over the next 14 days. During this incubation period the mosquitoes’ propensity to feed was assayed for each isolate by offering a feeding stimulant at the side of the cage and recording the number probing.

**Results and conclusions:**

Fungal isolates showed a range of virulence to *A. stephensi* with some causing >80% mortality within 7 days, while others caused little increase in mortality relative to controls over the study period. Similarly, some isolates had a large impact on feeding propensity, causing >50% pre-lethal reductions in feeding rate, whereas other isolates had very little impact. There was clear correlation between fungal virulence and feeding reduction with virulence explaining nearly 70% of the variation in feeding reduction. However, there were some isolates where either feeding decline was not associated with high virulence, or virulence did not automatically prompt large declines in feeding. These results are discussed in the context of choosing optimum fungal isolates for biopesticide development.

## Background

A number of recent reports suggest that the continued efficacy of malaria and mosquito vector control tools is threatened by drug and insecticide resistance [1-6]. The rise in pyrethroid resistance [5], the primary class of insecticide used in public health, is eroding efforts to control vectors with insecticide-treated nets (ITNs) and insecticide residual sprays (IRS) [7-11]. In the face of this resistance evolution, recent years have seen an increased interest in alternative vector control methods. One such approach is the use of fungal entomopathogens as novel active ingredients for use in biopesticides [12,13].

Fungi can be formulated and applied like chemical insecticides [14] and could be delivered through conventional approaches, such as IRS, or novel strategies such as resting targets [13,15], eave curtains [16] or odour baited traps [17]. Fungal pathogens have been shown to be effective against a range of vectors [18-22] including insecticide susceptible, resistant and multi-resistant mosquitoes [18] and to impact on the transmission potential of these vectors through effects which include reductions in feeding [12,18,21,23], fecundity [18,23], reduced flight capability [18], host location [24] and elevated metabolic rates [18]. In addition, recent work has shown that fungal shelf-life and post application persistence (important criteria for the active ingredient of any IRS product) on some surfaces can be comparable to chemical insecticides [Authors’ submitted manuscript].

These advances are encouraging but have not been without criticism [25], in part because fungi induce relatively slow mortality (often 7–14 days to reach >90% mortality) and this does not fit with the fast acting chemical insecticide target product profiles prescribed by the World Health Organization Pesticide Evaluation Scheme (WHOPES) [26]. However, rapid mortality is not always required for delivering effective malaria control [12,14,27-29] and there are potential benefits of a slow speed of kill for alleviating resistance evolution [29,30]. Furthermore, as indicated above, fungal infection can affect mosquito life history in multiple ways and virulence (speed of kill) is only one measure of pathogen impact.

The nature and extent of the lethal and pre-lethal effects of a fungal biopesticide will depend on the fungal isolate, the dose applied, the efficiency of dose transfer (affected by formulation, substrate and exposure time) and the temperature during fungal incubation in the vector [12,31-33]. To date around forty experimental studies have been reported examining some aspect of fungal infection on adult mosquito vectors [12,13,15-19,21-24,31-57]. These studies include multiple fungal isolates from eight genera and twenty-one species. In spite of this diversity, the majority of recent studies consider only a handful of fungal isolates and have tended to focus on virulence alone. This study evaluated the virulence of 17 isolates of the entomopathogenic fungi *Beauveria bassiana*, *Metarhizium anisopliae*, *M. acridum* and *Isaria farinosus* against the Asian vector mosquito *Anopheles stephensi*. In addition these isolates ability to affect feeding propensity and the relationship between virulence and alterations in feeding behaviour following fungal infection was assessed. These results are discussed in the context of making rational choices for malaria control products.

## Methods

### Fungal preparation and formulation

Fungal isolates (Table 1) were selected from a collection of isolates known to infect a diversity of insect taxa (including Diptera) and hence are reasonable candidates to infect *Anopheles* mosquitoes.

**Table 1:** Species, country of origin and the original host the fungal isolate was collected from (where known) for each of the isolates used in this study

**Fungal isolate**	**Species**	**Host origin**	**Country of origin**
**Bb01**	*Beauveria bassiana*	Coleoptera: Chysomelidae	USA
**Bb02**	*Beauveria bassiana*	*Musca domestica* (Diptera: Muscidae)	USA
**Bb03**	*Beauveria bassiana*	*Delia radicans* (Diptera: Delphacidae)	Canada
**Bb04**	*Beauveria bassiana*	*Nilparvata lugens* (Hemiptera: Delphacidae)	Solomon Islands
**Bb05**	*Beauveria bassiana*	*Musca domestica* (Diptera: Muscidae)	USA
**Bb06**	*Beauveria bassiana*	*Musca autumnalis* (Diptera: Calliphoridae)	France
**Bb07**	*Beauveria bassiana*	Soil sample	Australia
**Ma01**	*Metarhizium acridum*	*Ornithacris cavroisi* (Orthorptera: Acrididae)	Niger
**Ma02**	*Metarhizium robertsii*	*Curculio caryae* (Coleoptera: Curculionidae)	USA
**Ma03**	*Metarhizium robertsii*	*Curculio caryae* (Coleoptera: Curculionidae)	USA
**Ma04**	*Metarhizium robertsii*	*Curculio caryae* (Coleoptera: Curculionidae)	USA
**Ma05**	*Metarhizium anisopliae*	*Busseola fusca* (Lepidoptera: Noctuidae)	Kenya
**Ma06**	*Metarhizium anisopliae*	*Aedes triseriatus* (Diptera: Culicidae)	USA
**Ma07**	*Metarhizium anisopliae*	*Inopus rubriceps* (Diptera: Stratiomyidae)	Australia
**Ma08**	*Metarhizium brunneum*	*Carpocapsa pomonella* (Lepidoptera: Olethreutidae)	Austria
**Ma09**	*Metarhizium robertsii*	*Conoderus* sp. (Coleoptera: Elateridae)	USA
**If01**	*Isaria farinosus*	Diptera: Tachinidae	Poland

Conidia were harvested from slopes or plates to make a spore suspension of approximately 1×10^6^ conidia ml^–1^ in sterile 0.05% w/v Tween 80 (Sigma) in distilled water. One ml of this suspension was then used to inoculate 75 ml sterile liquid medium culture medium (4% d-Glucose, 2% yeast extract [Oxoid, UK] in tap water), in 250 ml Erlenmyer flasks. Flasks were incubated on a rotary shaker (160 rpm) at 24°C for 3 days.

Barley flakes (Bobs Red Mill, Milwaukie, Oregon, USA) were weighed into mushroom spawn bags (Unicorn, Garland, Texas, USA), 1 kg per bag and 600 ml tap water was added and the contents mixed by hand to ensure even absorption of the water. The spawn bags were then placed inside autoclave bags for protection and autoclaved for 30 min at 121°C. Once cool, the bags were inoculated under aseptic conditions with 75 ml of the 4-day old liquid medium plus 75 ml of sterile water to achieve a final moisture content of approximately 48%. The inoculated bags were carefully massaged to ensure even distribution of the inoculum. The bags were then sealed and incubated on shelves for 10 days at 24°C. Following incubation, the bags were opened in a reverse flow cabinet (Labconco, USA) and the contents transferred to brown paper bags for drying. The paper bags were placed in a dehumidified room for 4 days (24-30°C), until the sporulated substrate reached <20% moisture content. The conidia were then harvested from the barley flakes using a Mycoharvester (Acis Manufacturing, Devon, UK). The harvested conidia were placed in glass dishes and further dried in a desiccator over dry silica gel at 24°C. Once the conidia powder reached 5% moisture content, a small sample was taken for quality analysis and the remaining powder was sealed in foil laminated sachets with a small sachet of silica gel and stored at 5°C until use.

To test the viability of conidia in the formulation prior to spraying, 0.5 ml of the ‘stock’ was taken and diluted in pure Isopar M to a concentration of approximately 1×10^7^ conidia ml^-1^. One drop of this suspension was transferred onto SDA in 6 cm diameter Petri plates using a microspatula and spread evenly over the surface of the agar. Three plates were prepared for each isolate formulation and incubated at 25°C for 20 hr. After incubation, the conidia on the agar surface were examined under a compound microscope (at 500× magnification). Conidia were counted as germinated if a germ was visibly protruding from the conidium and all conidia in each field of view were assessed. A total of at least 300 conidia were counted per plate and viability was calculated as a percentage of the total.

### Mosquito rearing

*Anopheles stephensi* were reared under standard insectary conditions at 27°C, 80% humidity and 12 L: 12 D photo-period. The colony has been at Penn State for four years and was obtained from National Institute of Health where it has been in culture for at least a further ten years. Eggs were placed in plastic trays (25 cm × 25 cm × 7 cm) filled with 1.5 l of distilled water. To reduce variation in adult size at emergence, larvae were reared at a fixed density of 400 per tray. Larvae were fed Liquifry for five days and then Tetrafin fish flakes. From approximately two weeks after egg hatch, pupae were collected daily and placed in emergence cages. The adults that emerged were fed *ad libitum* on a 10% glucose solution. All experiments used three-to-five day old adult female mosquitoes.

### Virulence assays

Application of fungal suspension to the test substrates (cardboard pots) was carried out following a well-established methodology [12,39,40]. In brief the fungal conidia were formulated in a mix of mineral oils (80% Isopar M: 20% Ondina 22) similar to that described previously [12] and the concentration adjusted to give 1×10^9^ conidia ml^-1^. Spray applications employed a hand-held artist’s air-brush, which produced an aerosol of the formulation from a 25 ml glass reservoir attached to the spray nozzle. Each waxed cardboard challenge pot was opened and attached flat to the centre of the 1 m^2^ vertical spray zone within a laminar-flow hood. 20 ml of suspension was sprayed evenly from a distance of 25 cm across the entire spray zone providing a theoretical dose (see 39) of 2×10^6^ conidia cm-^2^. This dose is not a maximal operational dose or the large experimental dose and application rates that have shown very rapid mortality previously [18], but should represent a discriminating dose sufficient to distinguish between isolates on the basis of intrinsic variation in virulence. Pots were reassembled and left to dry for 24 h. 30 adult female mosquitoes were then transferred to each pot and left in place for 6 h before being removed to standard mosquito cages (30×30×30 cm) where they were maintained on an *ad libitum* supply of glucose water at 26 (±1)°C and 85 (±10)% RH. Mortality was recorded daily for 14 days after exposure. Each isolate was applied to 4 replicate pots and paired to equivalent control pots treated with blank formulation only.

### Feeding propensity assays

Feeding propensity was assessed using an established methodology [18]. Mosquitoes were offered a feeding stimulus comprising a 250 ml flask filled with hot tap water (~35-40°C) and covered socks worn recently by with one of the investigators (SB). This stimulus provides both a heat and odor cue and is a routine technique for sorting female from male *An. stephensi* for experimental purposes in the laboratory. The stimulus was placed adjacent to a mosquito cage and the proportion of mosquitoes recruiting to the cage wall and observed actively probing through the mesh over a 5-minute period, recorded. This assay was repeated daily for each replicate cage for each isolate and its respective controls.

### Statistical analysis

For all treatments median survival times were analysed using a Kaplan-Meier survival analysis in SPSS for Mac (v.19) with significant differences between doses and/or treatments estimated using a Log Rank Test. To examine feeding behaviour, repeated measures ANOVAs were performed on the mean proportion of mosquitoes attempting to feed each day from day 1 after exposure until mortality had reached 80%, or if this level was not achieved, then by the end of day 14. To determine the LT_80_ (the time to 80% mortality, which is the minimum mortality considered acceptable by WHOPES [42]) Weibull functions were fitted to the survival data:

(1) $$S=\exp\left[-\left(\text{A}/\text{G}\right)t^{\text{G}}\right]$$

where S is proportional survival, A and G are best-fit parameters and *t* is time.

## Results

### Survival

**Figure 1:**
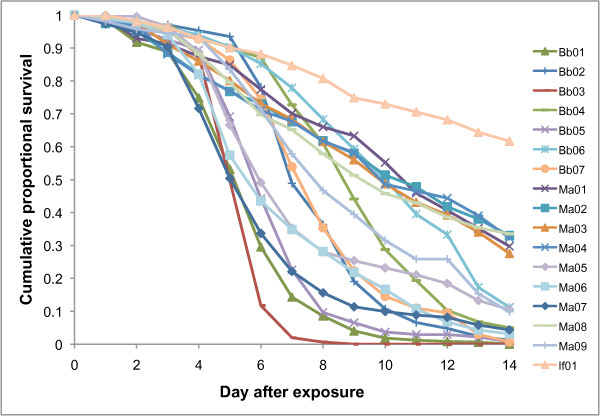
**Cumulative proportional survival of *Anopheles stephensi *exposed to different isolates of entomopathogenic fungi.** Mosquitoes were exposed to isolates of *Beauveria bassiana*, *Metarhizium* spp. and *Isaria farinosus* and their survival followed for fourteen days following exposure. For ease of comparison the figure omits the daily error bars and also excludes the control treatments paired to each of the 17 isolates.

**Figure 2:**
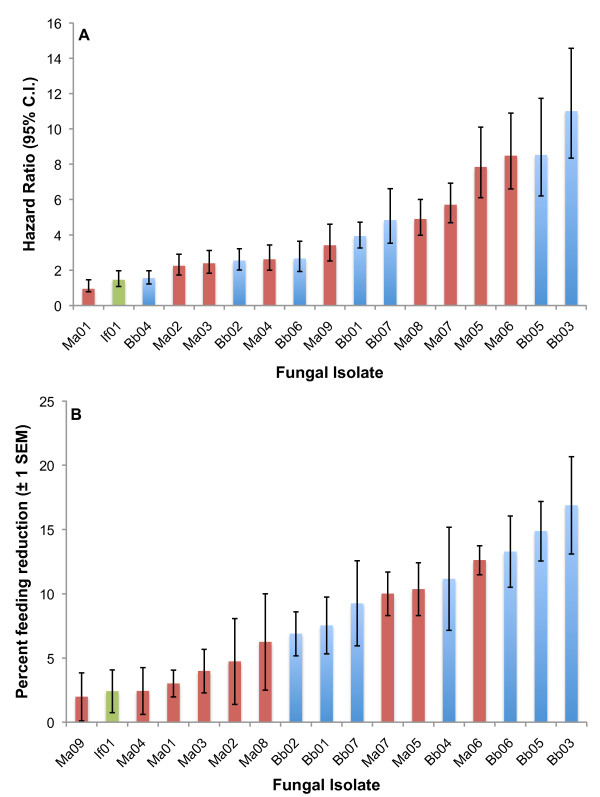
**Rank order of A) fungal virulence and B) impact on feeding propensity for 17 entomopathogenic fungal isolates.** Virulence is shown as the hazard ratio for each isolate estimated from a Cox regression. Impact on feeding propensity is based on the average reduction in feeding propensity until 80% of mosquitoes were dead, or until the end of the assay if mortality was less. Feeding propensity is corrected by subtracting the mean control feeding propensity over the same period. For each graph blue columns are *B. bassiana* isolates, red columns *Metarhizium* isolates and the green columns an *Isaria farinosus* isolate.

All but one of the fungal isolates tested caused significantly increased mortality of adult *A. stephensi* relative to the paired controls (Table 2) but there was large variation between isolates (Figure 1). Several isolates caused high levels of mortality with the most virulent (*B. bassiana* - Bb03) exhibiting a median survival time of 5 days and an LT_80_ of 5.8 days. Other isolates were less virulent, with some failing to achieve 80% mortality with the 14 days. The one isolate that did not differ from its control was one of the *M. anisopliae* isolates (Ma01), but in this case the controls showed much higher mortality than normal, possibly obscuring a treatment effect. In absolute terms, the single isolate of *I. farinosus* was the least virulent. There were no consistent differences between *B. bassiana* and *Metarhizium spp* isolates when the hazard ratios were ranked (Figure 2A).

**Table 2:** **Effects of a range of fungal isolates on survival of adult *Anopheles stephensi***

**Fungal isolate Number**	**Median lethal time (95% C.I.) days**	**Log rank statistic (Significance compared to controls)**	**Time to 80% mortality (± 1 SEM) days**	**Hazard ratio (95% C.I.)**
***Beauveria*spp.**				
**Bb01**	6.0 (5.81-6.19)	247.7 (*P* < 0.001)	7.3 (± 0.25)	3.93 (3.26-4.72)
**Bb02**	7.0 (6.64-7.36)	74.4 (*P* < 0.001)	6.0 (± 0.00)	2.54 (2.01-3.21)
**Bb03**	5.0 (4.80-5.20)	249.6 (*P* < 0.001)	9.3 (± 0.25)	11.0 (8.3-14.6)
**Bb04**	9.0 (8.53-9.47)	17.5 (*P* < 0.001)	11.3 (± 0.48)	1.55 (1.22-1.97)
**Bb05**	6.0 (5.66-6.34)	170.1 (*P* < 0.001)	8.0 (± 0.00)	8.53 (6.21-11.7)
**Bb06**	10.0 (9.04-10.96)	45.9 (*P*< 0.001)	13.3 (± 0.25)	2.65 (1.92-3.64)
**Bb07**	8.0 (7.54-8.46)	122.9 (*P* < 0.001)	10.3 (± 0.96)	4.83 (3.52-6.62)
***Metarhizium*spp.**				
**Ma01**	11.0 (10.21-11.79)	0.37 (*P* = 0.54)	Not achieved	0.94 (0.77-1.45)
**Ma02**	13.0 (11.72-14.28)	43.6 (*P* < 0.001)	Not achieved	2.24 (1.73-2.90)
**Ma03**	12.0 (10.47-13.53)	47.5 (*P* < 0.001)	Not achieved	2.38 (1.83-3.11)
**Ma04**	11.0 (9.80-12.20)	59.0 (*P* < 0.001)	Not achieved	2.16 (1.99-3.42)
**Ma05**	6.0 (5.75-6.25)	357.9 (*P* < 0.001)	11.3 (± 1.03)	7.85 (6.10-10.1)
**Ma06**	6.0 (5.56-6.44)	369.1 (*P* < 0.001)	10.0 (± 0.41)	8.48 (6.60-10.9)
**Ma07**	6.0 (5.71-6.29)	339.0 (*P* < 0.001)	8.0 (± 0.41)	5.70 (4.69-6.93)
**Ma08**	6.0 (5.75-6.25)	288.3 (*P* < 0.001)	8.5 (± 0.65)	4.89 (3.98-6.01)
**Ma09**	8.0 (7.17-8.83)	73.2 (*P* < 0.001)	13.0 (± 0.00)	3.41 (2.52-4.60)
***Isaria farinosus***				
**If01**	Not calculated	5.9 (*P* = 0.015)	Not achieved	1.45 (1.07-1.97)

Included are the median lethal time from Kaplain-Meier analysis and its significance relative to paired controls; time to 80% mortality (±1 standard error around the mean mortality across the replicate cages); and the hazard ratio estimated from a Cox regression which gives a measure of mortality risk relative to the controls.

### Feeding propensity

Declines in feeding propensity were clearly apparent in 10 of the isolates tested (Table 3). All *B. bassiana* isolates except Bb06 caused a significant reduction in feeding propensity (Table 3 and Figure 2B). The maximum reduction in feeding relative to controls on any one day was between 40 and 50% for several isolates. The *Metarhizium* isolates were much more variable with only 4 significantly reducing feeding propensity (Table 3 and Figure 2B). The *Isaria* If01 isolate had no impact on feeding propensity. Ranking the extent of feeding decline by comparing the average difference in feeding propensity between fungal exposed and control mosquitoes across the duration of the study (a conservative measure relative to the maximum) showed that *B. bassiana* isolates had a greater impact on feeding behaviour than *Metarhizium* isolates (Figure 2B).

**Table 3:** **Effects of a range of fungal isolates on the blood-feeding propensity of adult *Anopheles stephensi***

**Fungal isolate number**	**F statistic**^**a**^**(Significance level)**	**Overall percent feeding decline**^**b**^**(± 1 SEM)**	**Maximum percent feeding decline (± 1 SEM)**	**Percent biting risk at day 14**^**c**^**(difference to controls)**
***Beauveria*spp.**				
**Bb01**	20.9 (*P* = 0.004)	7.54 (±2.21)	17.8 (±6.51)	0 (−)*
**Bb02**	29.2 (*P* = 0.002)	6.89 (±1.71)	18.9 (±9.89)	0.6 (−11.5)
**Bb03**	294.3 (*P* < 0.001)	16.9 (±3.78)	48.8 (±18.7)	0 (−)*
**Bb04**	107.3 (*P* < 0.001)	11.2 (±4.01)	35.8 (±15.5)	0 (−)^§^
**Bb05**	29.4 (*P* = 0.002)	14.9 (±2.32)	38.3 (±13.5)	0 (−)^§^
**Bb06**	0.26 (*P* = 0.63)	13.3 (±2.77)	19.3 (±6.50)	10.7 (−31.9)
**Bb07**	6.26 (*P* = 0.046)	9.25 (±3.31)	42.8 (±23.7)	0 (−)^§^
***Metarhizium*spp.**				
**Ma01**	7.85 (*P* = 0.031)	7.54 (±4.3)	13.8 (±1.56)	23.1 (−5.12)
**Ma02**	2.18 (*P* = 0.19)	4.73 (±3.34)	19.1 (±9.17)	3.30 (−5.61)
**Ma03**	4.79 (*P* = 0.071)	3.98 (±1.70)	18.7 (±4.70)	3.63 (−5.28)
**Ma04**	1.09 (*P* = 0.34)	2.44 (±1.82)	11.9 (±4.76)	2.96 (−5.94)
**Ma05**	20.1 (*P* = 0.005)	10.4 (±2.06)	21.9 (±12.3)	7.06 (−55.3)
**Ma06**	78.4 (*P* < 0.001)	12.6 (±1.13)	41.8 (±7.12)	1.02 (−61.3)
**Ma07**	333.7 (*P* < 0.001)	10.0 (±1.69)	29.8 (±1.61)	2.52 (−44.3)
**Ma08**	3.92 (*P* = 0.095)	6.25 (±3.74)	8.31 (±3.81)	7.39 (−44.3)
**Ma09**	1.67 (*P* = 0.24)	1.98 (±1.87)	10.0 (±4.37)	7.56 (−35.1)
***Isaria farinosus***				
**If01**	2.16 (*P* = 0.19)	2.41 (±1.66)	9.96 (±3.44)	52.9 (−9.46)

Included are the main effects of treatment and their significance from the repeated measures ANOVA; the decline in feeding propensity averaged across the study period (±1 standard error around the mean decline in feeding propensity across replicates); the maximum feeding decline on any one day and the biting risk at day 14 (±1 standard error of the mean biting risk across replicates) around the mean mortality across the replicate cages). For both the latter measures the standard error around the mean. For further details see main text.

^a^ Generated from repeated measures ANOVA (see main text). All assays were comparisons of one isolate and the control with each replicated four times.

^b^ Calculated as the average proportion not feeding across the whole experiment until mortality had reached 80%, or if this level was not been reached, then across the whole 14 days.

^c^ Calculated as the product of the proportion of mosquitoes surviving at day 14 and the proportion responding to the feeding stimulant.

* Biting risk was zero because all mosquitoes had died in the fungal treatment.

§ Biting risk was zero because none of the remaining fungal exposed mosquitoes responded to the feeding stimulant.

### Correlation between survival and feeding

**Figure 3:**
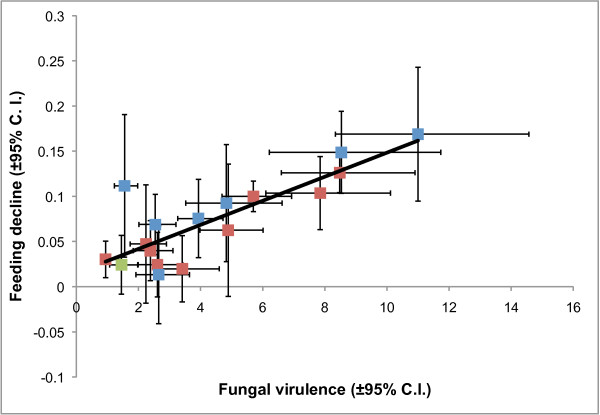
**The relationship between fungal virulence (the hazard ratio as in Table**2**and Figure**2A**) and the reduction in feeding propensity (Table**3**and Figure**2A**).** Fitted line shows a linear regression where Reduction in feeding propensity = (Hazard ratio × 0.015)+ 0.013. Blue squares are *B. bassiana* isolates, red squares *Metarhizium* isolates and the green square an *Isaria farinosus* isolate.

Regression analysis revealed a significant positive association between the proportion of mosquitoes not responding to the feeding stimulus and fungal virulence (measured as the hazard ratio) (*F*_1,14_ = 30.0, *P* < 0.001; R^2^ = 0.68; Figure 3). A couple of *B. bassiana* isolates that showed only low-to-intermediate virulence (Bb04 and Bb06) had higher impacts on feeding than their mortality hazard ratios might predict. There was no real evidence for the reverse pattern of high virulent isolates having negligible impacts on feeding. The one possible exception was *Metarhizium* isolate Ma01, which had no survival impact but still a significant effect on feeding propensity. However, as discussed above, the survival data for this isolate are slightly difficult to interpret as the control group suffered higher than expected mortality.

Biting risk (the product of mortality and feeding propensity) at day 14, which is approximately the time at which a mosquito could potentially transmit malaria if it acquired the parasite at its first blood feed, showed that all *B. bassiana* isolates had a large impact on transmission potential. For 5 out of the 7 isolates, biting risk was reduced to zero (and for one of these remaining isolates, it was practically zero). The *Metarhizium* isolates had a more mixed impact with biting risk greatly reduced relative to controls in five of the nine isolates tested but with reductions of between 35-61% only (Table 3). The single *Isaria* isolate caused only marginal declines in biting risk in line with its very modest virulence.

## Discussion

The simple lab assay screen revealed that all fungal isolates examined had some effect on adult *A. stephensi*, reducing either survivorship, feeding propensity, or both. There was no obvious difference between fungal species in terms of virulence (the *I. farinosus* isolate had very low virulence but with a sample size of only one it is not possible to say whether this was a strain effect or species effect). In general the more virulent isolates also had the greatest impact on feeding, although for the combined metric of ‘biting risk’, the *B. bassiana* isolates tended to come out on top with several reducing biting risk to zero by day 14. Increased doses or more effective dose transfer would likely bring forward this impact to within one or two feeding cycles [18].

Several other studies have demonstrated reduced feeding propensity in mosquito vectors following fungal infection [12,18,21,23]. This is potentially a very important pre- or sub-lethal effect as there can be no transmission without feeding, even if the vectors are alive. The mechanisms involved in the anti-feeding effects are unclear. It has been suggested previously that feeding reductions are due to resource competition within the host and/or mechanical damage of host tissue as the fungal hyphae proliferate [58,59]. Fungal pathogens appear to go through a period of very low replication after infection followed by rapid increase in biomass just prior to host death [39,60]. Such growth patterns could explain the reductions in feeding observed here, especially for the virulent isolates. In addition, entomopathogenic fungi are known to produce their own enzymes for converting the host’s stored sugars (e.g. trehalose) to glucose [61]. In phytophagous insects one of the feedback mechanisms implicated in decreasing motivation to feed involves a concentration gradient of glucose between the gut and haemolyph [62]. It is possible that fungal-induced changes in key nutrient gradients such as glucose could also play a role here [63]. Other (not mutually exclusive) mechanisms could include reduced olfactory sensitivity [24] or illness-induced anorexia in response to infection and immuno-stimulation [64]. Better understanding these mechanisms could help in screening or selecting isolates with stronger anti-feeding properties.

One interesting pattern revealed by this study is that isolates that induce more or less equivalent overall reductions in transmission potential can do so following very different daily mortality trajectories. Isolate Bb01 and Bb04, for example, both show zero biting risk at day 14 but Bb01 has an LT_80_ of 7.3 days while Bb04 has an LT_80_ of 11.3 days. Slower speed of kill potentially allows a higher proportion of lifetime reproductive output to be achieved and hence reduces selection pressure for resistance [14,29]. While no studies to date have shown resistance to insect fungal pathogens [65], evidence from other microbial agents used in insect control has shown resistance can evolve [66-68], and heritable genetic variation in susceptibility to *B. bassiana* has been demonstrated in *Drosophila*[69]. Given that the interest in alternative control tools is motivated by resistance against conventional insecticides, it could be valuable to evaluate potentially more ‘evolution proof’ isolates, such as Bb04, from the outset. An important note in this regard is that the number of isolates screened here represents a tiny sample of the available options. The USDA’s fungus collection (Agricultural Research Service Collection of Entomopathogenic Fungi - ARSEF) holds an estimated 2,000+ *B. bassiana* and 2,500+ *Metarhizium* spp. isolates. Other national and international collections add to the list. With some very simple selection criteria (in this case the isolates were known to infect more than one insect taxa) it appears relatively straightforward to identify isolates that can infect mosquitoes. Under the current test conditions, all 17 isolates had some impact and a couple of the *B. bassiana* isolates appeared as good or better (in terms of virulence and anti feeding effects) than the *B. bassiana* isolate (IMI39150) that has been the focus of numerous recent mosquito studies. Given the political controversies surrounding GM organisms, it might be worthwhile exploring the rich fungal diversity within existing collections to better define natural variation in infection phenotypes before embarking on novel genetic engineering strategies to artificially generate desirable traits [70].

In conclusion, this study adds to growing evidence that fungal pathogens could contribute to disease vector control and offer a rich resource of phenotypes to explore. A basic screen such as this is a very long way from an operational product. In addition to desirable transmission blocking properties, any fungal product has to be amenable to mass production, store well after being produced and persist well after application, as well as meet stringent criteria for human and environmental safety [4,31,71]. While precedents for developing fungal biopesticides for use in agriculture in Africa and Asia exist [14], one current barrier to wider acceptance in public health is the restrictive WHOPES criteria used to evaluate products for inclusion in vector control portfolios. The approach of emphasizing only those products that kill vectors rapidly and can persist after application for six months or more currently excludes a number of novel interventions, not just fungi [29,72-77]. Given the looming resistance crisis in malaria these criteria require reassessment, particularly where products are to be used in integrated vector management strategies where their effectiveness depends on the ‘sum of the parts’ and not simply their stand alone contribution [78]. It is important that regulatory frameworks are amenable to innovative research and development and do not inadvertently create barriers to ultimate commercialization [78].

## Competing interests

The authors declare that they have no competing interests.

## Authors’ contributions

SB, NEJ, AFR and MBT conceived and designed the experiments. SB and NEJ conducted the experiments. SB analysed the results. SB, MBT, AFR drafted the manuscript. All authors read and approved the final manuscript.
